# Assessment of radiation safety awareness among nurses in nuclear medicine departments

**DOI:** 10.7717/peerj.21109

**Published:** 2026-04-17

**Authors:** Abdullah Fahad A. Alshamrani, Faisal A. Alrehily, Mansour M. Alqahtani, Moawia Gameraddin, Majed Alharbi, Tasneem S.A. Elmahdi, Zeyad Awadh Alharbi, Tariq Zayd Almutairi, Osama Abdullah Al johani

**Affiliations:** 1 Department of Diagnostic Radiology Technology, College of Applied Medical Sciences, Taibah University, Madinah, Saudi Arabia; 2 Department of Radiological Sciences, College of Applied Medical Sciences, Najran University, Najran, Saudi Arabia; 3 Physics Department, College of Science, Jouf University, Sakaka, Saudi Arabia

**Keywords:** Radiation safety, Nuclear medicine, Nurses, Awareness, Radiation protection

## Abstract

**Background:**

Nuclear medicine is a critical specialty involving the use of radioactive materials for diagnostic and therapeutic purposes. Nurses working in these departments are frequently exposed to ionizing radiation, placing them at occupational risk. Despite the availability of safety guidelines, the extent of nurses’ awareness of and adherence to radiation protection protocols remains uncertain. This study was conducted to assess the level of radiation safety awareness among nurses in nuclear medicine departments across Saudi Arabia.

**Methodology:**

A cross-sectional survey was conducted from 19 January to 24 March 2025 using a structured, self-administered questionnaire distributed via online platforms. A total of 233 registered nurses participated and met the predefined inclusion criteria. The data were analyzed using SPSS, focusing on descriptive statistics and inferential tests to evaluate awareness, knowledge, and protective practices.

**Results:**

Most nurses demonstrated moderate-to-good general awareness of radiation safety. However, gaps were identified in specific areas, including knowledge of dose limits, use of personal dosimeters, and formal training. Notably, over half of the participants had not received structured radiation safety education, and adherence to protective practices, such as exposure time, distance, and shielding, was inconsistent. Institutional support was found to be present in many settings; however, protocol enforcement varied widely.

**Conclusion:**

The study reveals a need for enhanced, continuous radiation safety training and stronger institutional enforcement to ensure consistent protective practices among nurses in nuclear medicine. Improving awareness and compliance is essential for both staff safety and quality patient care.

## Introduction

In nuclear medicine (NM), radioactive materials are used to target specific biological pathways for diagnosing and treating diseases. Radiopharmaceutical agents are delivered to patients to evaluate organ function. Unlike traditional radiology, which focuses on anatomical structures, NM provides insights into physiological and metabolic processes through imaging techniques such as positron emission tomography (PET) and single-photon emission computed tomography (SPECT) (Cutler et al., 2021; Vaz et al., 2020).

NM departments in Saudi Arabia follow regulations set by the Saudi Food and Drug Authority (SFDA) and the Nuclear and Radiological Regulatory Commission (NRRC), both of which outline how teams should work together to maintain staff and patient safety in radiation environments. In daily practice, NM radiographers perform technical imaging tasks and prepare and administer radiopharmaceuticals under the supervision of a physician. These radiographers are also central to ensuring safety protocols are followed. In contrast, NM nurses are hands-on with patient care throughout all phases of each scan, from preparing, educating, and supporting patients to monitoring for side effects and managing emergencies when needed (Brown, 2012; Vijayakumar et al., 2006; Wang, Voss & Dolansky, 2021). Every hospital must appoint a Medical Radiation Safety Officer, such as a medical physicist or radiologist, to lead and monitor safety practices, provide staff training, and ensure policies are actively followed (Heston & Tafti, 2024).

However, many nurses may have limited awareness of radiation risks and the safety measures needed to minimize exposure. Nurses may be harmed by increased occupational hazards resulting from a lack of understanding of radiation safety procedures (Choudhary, 2018). Training programs and guidelines exist for radiation protection; however, formal programs providing education on radiation hazards and protection within nursing curricula could improve radiation safety awareness among nurses (Salih et al., 2023).

Nurses must have background knowledge of radiation risks to ensure that they are fully aware of the potential risks of exposure and equipped to take the necessary precautions to protect themselves, their patients, and their colleagues. Without proper education, nurses may unknowingly expose themselves to higher levels of radiation, increasing the risk of long-term health issues such as cancer, genetic mutations, and other radiation-related illnesses (Choudhary, 2018; Kamiya et al., 2015).

Proper training equips nurses with the knowledge to follow safety protocols, including the As Low As Reasonably Achievable (ALARA) principle; furthermore, it ensures that nurses understand how to correctly use personal protective equipment (PPE), such as lead aprons, gloves, and radiation-monitoring devices, which are essential for reducing exposure (Kim, 2018; Hendee & Marc Edwards, 1986).

Nurses who are well-trained in radiation safety can respond quickly and effectively to emergencies (Veenema & Thornton, 2015), such as spills, leaks, or accidental exposure. Their knowledge helps them control the situation, prevent contamination, and reduce health risks.

Existing radiation safety training programs may not provide nurses with sufficient education on the specific challenges of NM safety (Yunus et al., 2014). These programs are usually aimed at radiologic technologists and physicians rather than focusing on the unique duties of nurses engaged in direct patient care (Donaldson, 2020). Furthermore, many training programs do not include hands-on learning approaches, such as practical simulations or real-world case studies, which are important for strengthening nurses’ ability to apply their theoretical knowledge (Johnson, Mu & Sawyer, 2012; Vassileva et al., 2022; Rodrigues et al., 2024). As a result, current radiation safety education often falls short of preparing nurses for the complex, real-time decisions they face in NM practice, where translating theoretical principles into safe bedside care is critical. Despite multiple international reports on radiation safety and awareness, there is limited published evidence specifically addressing radiation protection knowledge and practices among NM nurses in Saudi Arabia, highlighting a clear local knowledge gap.

This study was conducted to assess nurses’ radiation awareness in NM departments in Saudi Arabia, specifically regarding their knowledge of safety practices and exposure risks. Furthermore, we aimed to identify knowledge gaps and areas needing improvement to guide the development of more comprehensive and targeted educational programs.

### Literature review

A literature review of numerous studies revealed that even low doses of ionizing radiation can increase the risk of developing certain cancers, particularly with cumulative exposure over time (Linet et al., 2010; Azmoonfar et al., 2024). For example, short-term exposure to doses of 10 to 50 mSv or long-term exposure to 50 to 100 mSv has been associated with an increased risk of some cancers (Wrixon, 2008). Nurses working in NM face occupational hazards due to continuous exposure to ionizing radiation, which can accumulate over their careers and increase the risk of developing radiation-induced diseases (Linet et al., 2010; Azmoonfar et al., 2024).

Despite these risks, studies have shown that nurses working in NM often lack essential knowledge of radiation protection practices. Although some nurses possess a basic understanding of radiation safety, significant gaps remain in their knowledge and application of radiation protection protocols (Rahimi et al., 2021; Alotaibi et al., 2015; Uçan & Çınar, 2022). For example, Rahimi et al. (2021) found that although Malaysian nurses scored relatively well on general radiation safety knowledge, their understanding of radiation physics and regulatory guidelines was insufficient, suggesting a need for more focused educational efforts. Similarly, Alotaibi et al. (2015) reported a concerning lack of awareness regarding radiation risks among nurses in Kuwait’s NM departments. Uçan & Çınar (2022) noted that many nurses did not receive adequate radiation safety training during their academic or professional careers, resulting in a knowledge gap compared with physicians and radiologic technologists. Thus, the importance of ongoing training programs to enhance nurses’ understanding of radiation safety is clear.

Supporting this, a recent study demonstrated that nurses who attended radiological preparedness training were more willing and better equipped to respond to nuclear events (Ochiai et al., 2022). Similarly, the findings of a study by Yunus et al. (2014) supported ongoing professional development programs to refresh nurses’ knowledge about radiation safety, increasing their overall awareness.

Experience also contributes significantly to nurses’ awareness levels. Alzubaidi, Al Mutairi & Alakel (2017) found that nurses with more years of experience demonstrated a better attitude towards ionizing radiation, reinforcing the idea that practical experience improves awareness and safety practices in NM.

## Materials & Methods

This cross-sectional study was conducted to assess the level of radiation safety awareness among nurses working in both public and private sector institutions across 68 NM departments in Saudi Arabia. Data were collected from 19 January 2025 to 24 March 2025. Nurses’ knowledge, attitudes, and practices related to radiation safety were assessed.

A total of 233 registered nurses participated and met the predefined inclusion criteria. Participants were selected by convenience sampling from multiple institutions. The questionnaire applied was a structured, self-administered tool comprising 24 items designed to assess nurses’ awareness across various domains, including demographic information, general awareness, radiation safety knowledge, radiation protection practices, and training and awareness. The survey employed two main scales: a frequency scale (*e.g.*, Never to Always) to measure how often certain practices or behaviors are performed, and a quality/satisfaction scale (*e.g.*, Poor to Excellent and Yes or No) to evaluate perceptions of knowledge or training quality. Furthermore, the questionnaire used closed-ended questions, in which respondents chose from a list of options. These qualitative responses were converted to numerical values for analysis, enabling meaningful comparisons and insights into awareness levels and practices. The questionnaire used was adapted from previously validated published studies and then reviewed by a panel of three experts in NM field to ensure content validity (Rahimi et al., 2021; Alotaibi et al., 2015; Ahmed et al., 2016; Alyami et al., 2022).

The questionnaire was created using Google Forms and distributed *via* email and to medical nursing communities on social media platforms (Telegram, WhatsApp, and X (formerly Twitter)). All participants were recruited voluntarily who received the research aim and objectives and signed the consent form electronically to participate in this study and the participants were anonymous.

The target population comprised registered nurses working in NM departments in Saudi Arabia. The inclusion criteria were as follows: (1) registered nurses with a valid nursing license from the Saudi Commission for Health Specialties (SCFHS); (2) nurses currently working in or with previous experience working in the NM department; and (3) nurses willing to participate in the study. Nurses who did not complete the questionnaire, refused to provide informed consent, or worked in other departments were excluded.

The data were analyzed using Statistical Package for the Social Sciences (SPSS) software (ver. 29.0.0.0, IBM Corp., Armonk, NY, USA). Descriptive statistics (mean and percentage), chi-square tests, the chi-square statistic (*χ*^2^) and degrees of freedom (df) were used to assess awareness levels and identify influencing factors. It was considered significant when *p*-values were less than 0.05. Ethical approval was obtained from the Deanship of Postgraduate Studies and Scientific Research at Taibah University (Ethical Application Ref: 2025/198/301 RAD). All investigations were conducted in accordance with the Declaration of Helsinki and all applicable standards and laws.

## Results

### Demographic data

Most respondents were adults aged 26–35 and 36–45, accounting for 68.7% of the total sample. Most participants were female (68.2%), 57.1% held a bachelor’s degree, and 36.1% held a diploma qualification. Regarding professional experience, the distribution was relatively balanced: 24.0% had less than 1 year of experience, 29.6% had 1–3 years, 20.2% had 4–6 years, and 26.2% had more than 6 years. The demographic characteristics of the study respondents are shown in Table 1, which summarizes the distribution of participants across variables such as sex, age, educational level, and experience.

**Table 1:** Demographic characteristics of the study respondents.

**Variables**	**Frequency**	**Percent %**
**Gender**		
Female	159	68.2%
Male	74	31.8%
**Age groups**		
Under 25	56	24%
26–35 years	76	32.6%
36–45 years	84	36.1%
46–55 years	15	6.4%
>55 years	2	0.9%
**Qualification**		
Diploma	84	36.1%
Bachelor	133	57.1%
Master	15	6.4%
Ph.D.	1	0.4%
**Experience**		
Less than 1 year	56	24%
1–3 year	69	29.6%
4–6 year	47	20.2%
>6 years	61	26.2%

### General awareness

**Figure 1:**
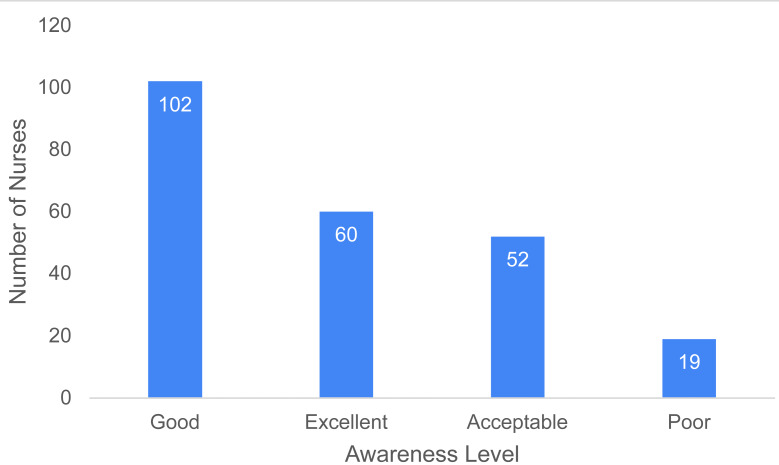
Distribution of participants by their awareness of the long-term health effects of radiation exposure.

Regarding awareness of the long-term health effects of radiation exposure, 102 participants (43.8%) rated their awareness as “Good”, while 60 (25.8%) rated it as “Excellent”. In contrast, 52 participants (22.3%) rated their awareness as “Acceptable”, while 19 (8.2%) rated it as “Poor” (Fig. 1).

**Figure 2:**
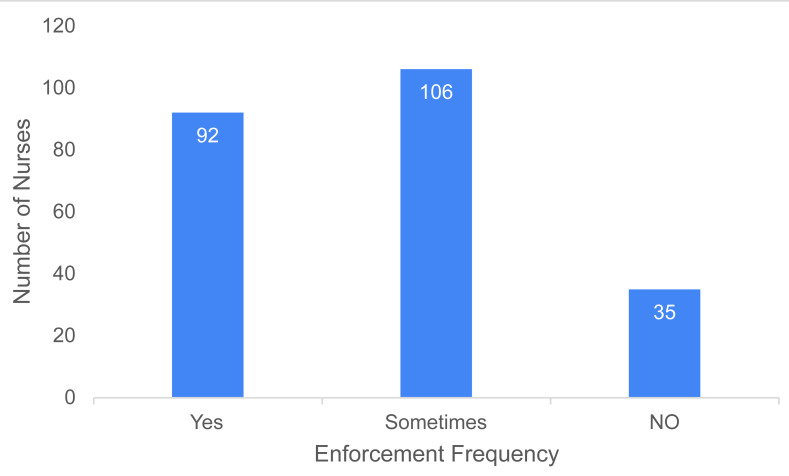
Distribution of participants according to the strictness of radiation safety protocol enforcement at their workplaces.

The enforcement of radiation safety protocols in the department varied among participants. Most of the participants (106; 45.5%) reported that the protocols were enforced “Sometimes”, while 92 (39.5%) felt they were enforced “Yes strictly”. In contrast, 35 participants (15.0%) believed that the protocols were not enforced sufficiently (Fig. 2).

Ease of access to radiation protection equipment, such as lead aprons and shielding, was reported by most participants (184; 79.0%) as readily available, while 49 (21.0%) indicated difficulty accessing such equipment.

The level of institutional support for maintaining radiation safety standards was rated as adequate by 169 participants (72.5%), whereas 64 (27.5%) reported insufficient support from their institutions.

The effectiveness of radiation protection measures in reducing exposure risks was rated as “Very effective” by 102 participants (43.8%) and “Moderately effective” by 87 (37.3%). In contrast, 43 participants (18.5%) considered the measures “Slightly effective”, and 1 (0.4%) rated them as “Not effective”.

**Figure 3:**
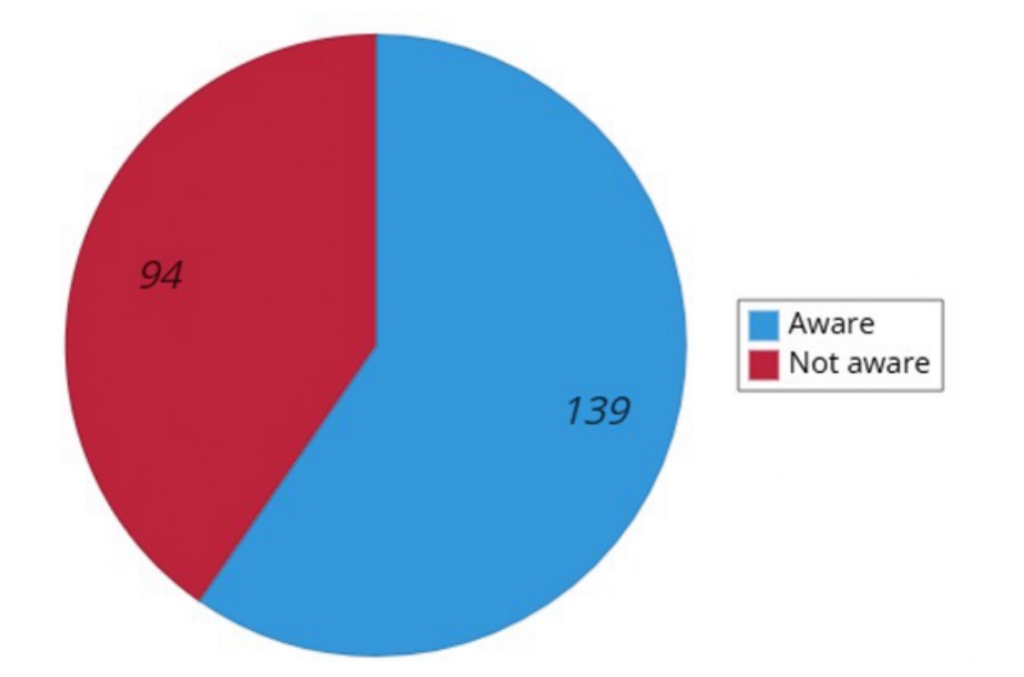
Distribution of participants according to their awareness of radiation dose limits.

Awareness of radiation dose limits among nurses working in the NM department varied: 139 participants (59.7%) reported being aware of the limits, while 94 (40.3%) reported not being aware of them (Fig. 3).

### Radiation safety knowledge

Regarding formal training on radiation safety related to NM, 119 participants (51.1%) reported not having received such training, while 114 (48.9%) indicated that they had received formal training specific to NM protocols.

Familiarity with the ALARA principle was reported by 185 participants (79.4%), while 48 (20.6%) indicated they were not familiar with it.

Knowledge of radiation safety practices in nuclear medicine was rated as “Good” by 95 participants (40.8%) and “Fair” by 74 (31.8%). Additionally, 53 participants (22.7%) rated their knowledge as “Excellent”, while 11 (4.7%) rated it as “Poor”.

Consultation of radiation safety guidelines or protocols varied among participants: approximately 85 (36.5%) reported consulting them “Frequently”, 73 (31.3%) stated they do so “Occasionally”, 72 (30.9%) indicated they “Always” consult the guidelines, and 3 (1.3%) reported “Never” consulting them.

Confidence in following radiation safety protocols varied among participants: 83 (35.6%) reported being “confident”, 71 (30.5%) were “very confident”, 56 (24.0%) were “somewhat confident”, and 23 (9.9%) were “not confident”.

### Radiation protection practices

The study revealed several areas where workplace radiation protection practices could be enhanced. A total of 55 participants (23.6%) identified imaging rooms as areas needing improvement, with an equal number (55, 23.6%) expressing uncertainty. Additionally, 51 participants (21.9%) highlighted the handling and storage of radioactive materials as a key area for improvement, while 40 (17.5%) reported no need to change radiation protection practices. Furthermore, 32 participants (13.7%) suggested improvements in the patient preparation areas.

Most of the 233 participants reported using various radiation protection measures. Specifically, 156 (67.0%) stated that they regularly use lead shielding, while 152 (56.2%) indicated they maximize their distance from the radiation source. Additionally, 138 participants (59.2%) reported limiting their exposure time, and 108 (46.4%) mentioned using dosimeters. Only four participants (1.7%) stated that they do not use any radiation protection measures.

Of 233 participants, 84 (36.1%) reported “Always” using time, distance, and shielding as radiation protection strategies during procedures, while 68 (29.2%) stated they “Frequently” apply these measures. Additionally, 60 participants (25.8%) indicated they “Occasionally” use them, 18 participants (7.7%) indicated that they “Rarely” use them, and 3 (1.3%) stated that they “Never” use them.

### Training and awareness

Of 233 participants, 211 (90.6%) indicated a need for more training in radiation protection, while 22 (9.4%) did not.

Regarding preferred methods for receiving radiation safety training, 125 participants (53.6%) favored workshops, while 123 (52.8%) preferred online courses. Additionally, 97 participants (41.6%) preferred seminars, 92 (39.5%) preferred on-the-job training, and 58 (24.9%) preferred printed guidelines.

When asked about the frequency of radiation protection training, 90 participants (38.6%) indicated that it should be offered annually, while 70 (30.0%) believed it should be offered every 2 years. Additionally, 56 participants (24.0%) suggested that it should be provided only when necessary, and 17 (7.4%) stated that it should be offered every 5 years.

Regarding their current level of knowledge about radiation protection, 142 participants (60.9%) felt their knowledge was sufficient, while 91 (39.1%) expressed a lack of confidence in their knowledge.

The main source of radiation protection knowledge for 79 participants (33.9%) was on-the-job training, while 76 participants (32.6%) reported receiving training during formal education. Additionally, 62 (26.6%) indicated that colleagues or supervisors were their source of knowledge, and 16 (6.9%) reported relying on personal research.

Concerning the use of personal dosimeters during NM procedures, 106 participants (45.5%) stated they use them “Always”, while 57 (24.5%) said they “Frequently” use them. Additionally, 42 (18.0%) reported using them “Sometimes”, and 28 (12.0%) indicated they “Never” use them.

Many participants had limited awareness of dose limits and lacked formal training in radiation safety. Nurses across all experience groups strongly agreed on the need for more radiation protection training, with over 87% in each group expressing this view. Awareness of dose limits was highest among mid-career staff (4–6 years experience) and was noticeably lower in the most experienced group. Formal training was uneven, particularly among those with more than 6 years of experience. Confidence in having sufficient knowledge was greatest among less experienced and mid-career nurses (Table 2). No significant association was observed between years of experience and dose limits, formal training and level of radiation protection knowledge with *P*-value (*χ*^2^ (3) = 6.88, *p* = 0.07), (*χ*^2^ (3) = 7.10, *p* = 0.06) and (*χ*^2^ (3) = 6.83, *p* = 0.07) respectively.

**Table 2:** Radiation safety knowledge and training among NM nurses by experience level.

**Question**	**Years of experience**	**Yes**	**No**	**Total**	**Mean**	*χ* ^2^	**df**	***P*-value**
Are you familiar with the dose limits for nurses working in nuclear medicine?	Less than 1 year	36	20	56	0.64	6.88	3	0.07^•^
	1–3 years	43	26	69	0.62			
	4–6 years	32	15	47	0.68			
	More than 6 years	28	33	61	0.46			
Have you received any formal training on radiation safety protocols specific to nuclear medicine?	Less than 1 year	28	28	56	0.50	7.10	3	0.06^•^
	1–3 years	41	28	69	0.59			
	4–6 years	23	24	47	0.49			
	More than 6 years	22	39	61	0.36			
Do you think more training in radiation protection is necessary for nurses in nuclear medicine?	Less than 1 year	51	5	56	0.91	1.02	3	0.79^•^
	1–3 years	64	5	69	0.93			
	4–6 years	41	6	47	0.87			
	More than 6 years	55	6	61	0.90			
Do you feel your current level of radiation protection knowledge is sufficient?	Less than 1 year	39	17	56	0.70	6.83	3	0.07^•^
	1–3 years	35	34	69	0.51			
	4–6 years	33	14	47	0.70			
	More than 6 years	35	26	61	0.57			

**Notes.**

* Mean is calculated as proportion answering “Yes” (Yes = 1, No = 0).

• Not Significant “*P*-value > 0.05”.

There was noticeable variation in the enforcement of radiation safety protocols, especially among less experienced staff, who reported inconsistent enforcement. A strong majority agreed on the need for more training, with annual refreshers preferred. Despite this, over half felt that their radiation protection knowledge was adequate, relying heavily on colleagues and on-the-job training. Usage of personal dosimeters varied with experience, highlighting opportunities to improve monitoring practices (Table 3). There was a significant association between years of experience and enforcement of radiation safety protocols, source of radiation protection knowledge and use of personal dosimeters with *P*-value (*χ*^2^ (6) = 40.37, *p* < 0.001), (*χ*^2^ (9) = 37.63, *p* < 0.001) and (*χ*^2^ (9) = 44.84, *p* < 0.001), respectively.

**Table 3:** Radiation safety protocols and training among NM nurses by experience level.

**Do you believe that radiation safety protocols in your department are strictly enforced?**							
**Years of experience**	**Yes**	**Sometimes**	**No**	*χ* ^2^	**df**	***P*-value**
**Less than 1 year**	32	19	5	40.37	6	<0.001
**1–3 years**	20	25	24			
**4–6 years**	17	25	5			
**More than 6 years**	23	37	1			

**How frequently do you think radiation protection training should be provided?**							
**Years of experience**	**Annually**	**Every 2 years**	**Every 5 years**	**Only when necessary**	*χ* ^2^	**df**	***P*-value**
**Less than 1 year**	38	11	2	5	53.61	9	<0.001
**1–3 years**	32	17	3	17			
**4–6 years**	7	14	4	22			
**More than 6 years**	13	28	8	12			

**What is the main source of your radiation protection knowledge?**							
**Years of experience**	**Colleagues or supervisors**	**Formal education**	**On-the-job training**	**Personal research**	*χ* ^2^	**df**	***P*-value**
**Less than 1 year**	10	28	11	7	37.63	9	<0.001
**1–3 years**	21	19	22	7			
**4–6 years**	21	13	12	1			
**More than 6 years**	10	16	34	1			

**Do you use personal dosimeters during nuclear medicine procedures?**							
**Years of experience**	**Always**	**Frequently**	**Never**	**Sometimes**	*χ* ^2^	**df**	***P*-value**
**Less than 1 year**	30	8	10	8	44.84	9	<0.001
**1–3 years**	36	11	15	7			
**4–6 years**	22	18	2	5			
**More than 6 years**	18	20	1	22			

## Discussion

Exposure to ionizing radiation is a potential hazard faced by healthcare workers in NM departments, particularly nurses who interact with patients during nuclear imaging and treatment procedures. Therefore, assessing nurses’ radiation safety awareness is crucial to ensure adherence to protective measures and minimize unnecessary radiation exposure, ultimately benefiting both their health and patient safety.

The results of this study indicate that radiation safety awareness among nurses in NM departments is generally moderate to good, yet important gaps remain, particularly in advanced safety measures and practical application. While most participants demonstrated basic knowledge of radiation protection principles, their limited familiarity with more specialized aspects of radiation safety mirrors earlier work by Yunus et al., (2014), who found that nurses in NM settings often lack focused training on detailed protection strategies. Building on this, Alyami et al. (2022) showed that regular participation in radiation safety courses is associated with higher awareness levels, and a similar pattern emerged in the present study: nurses who had received NM-specific radiation safety training exhibited better knowledge than those without such training. However, consistent with Al-Shahrani et al., (2024), structural barriers appear to constrain the translation of knowledge into practice, as reflected in suboptimal use of PPE and monitoring devices despite nominal equipment availability. This discrepancy suggests that issues such as accessibility of protective tools, workflow constraints, or insufficient reinforcement of safety culture may be undermining effective implementation of radiation protection protocols, highlighting the need for institutions to prioritize not only training provision but also the practical integration of safety resources into daily clinical practice.

Plouss & Efstathopoulos (2016) highlighted the critical role of the work environment in shaping nurses’ compliance with radiation safety standards, showing that departments with clear, consistently implemented policies achieve markedly higher adherence than those with inconsistently applied guidelines. The pattern observed in the present study is similar: only a minority of nurses perceived institutional support and enforcement as consistently strong, while many reported that policies were applied only intermittently. Rather than reflecting knowledge deficits alone, this variability suggests that organizational factors such as leadership commitment to safety, clarity of procedures, and routine monitoring are key drivers of day-to-day compliance. A robust safety culture, supported by visible managerial engagement and regular feedback, therefore appears essential for translating awareness into sustained safe practice. Although this study did not directly assess the patient safety outcomes, existing literature suggests that inadequate radiation safety awareness among healthcare workers may increase the risk of occupational exposure and compromise safe clinical practice; therefore, the observed awareness gaps should be interpreted as potential concerns rather than direct indicators of patient harm.

Our findings also echo international literature showing that continuous education and structured training programmes are central to improving radiation safety awareness among nurses in NM and related settings. Studies from Kuwait, China, and other regions have consistently reported substantial knowledge gaps and have called for formal, recurring training tailored to the specific risks and procedures of NM practice (Salih et al., 2023; Alotaibi et al., 2015; Xie et al., 2025). Recent reviews of radiation literacy among healthcare professionals further indicate that these gaps persist despite the availability of guidelines, underscoring the need for targeted educational interventions and integration of radiation protection content into both pre-registration curricula and ongoing professional development (Xie et al., 2025; Massaquoi et al., 2025). Together, these observations suggest that meaningful improvement requires a combined approach: strengthening institutional safety culture and policy enforcement while simultaneously providing high-quality, context-specific training for nursing staff.

Although nurses working in NM departments might be expected to have uniformly high awareness of radiation safety, this study revealed marked variability in awareness levels. Differences in initial training and the lack of structured, continuous educational programmes likely contribute to these disparities, as earlier work has shown that without regular reinforcement, adherence to safety procedures declines even when staff possess adequate theoretical knowledge (Brown, 2012). The strong perceived need for additional radiation protection training among participants in this study, particularly those with less experience, reinforces the idea that one-off courses are insufficient. The widespread preference for annual refresher courses suggests that nurses recognise the importance of ongoing education to maintain safe practice.

At the same time, the findings indicate that attendance at training does not automatically translate into high awareness or confidence. A substantial proportion of nurses who had received formal radiation safety training still did not rate their knowledge at the highest level, and many reported only moderate confidence in applying safety protocols in daily work. This pattern points to potential shortcomings in the design and delivery of existing training, which may focus heavily on theory while providing limited opportunities for supervised practice or skills rehearsal. Consistent with evidence that practical, scenario-based training is more effective than didactic lectures for changing safety-related behaviours (Ochiai et al., 2022). Our results highlight the need to integrate hands-on components, simulation, and workplace-based reinforcement into radiation safety education so that theoretical knowledge is translated into confident and consistent adherence to protection procedures. Effective models include mandatory workshops, online courses, and simulation-based training, which have been shown to significantly increase knowledge retention and promote safe practices (Rodrigues et al., 2024; Jeyasugiththan et al., 2023). For example, face-to-face group training sessions tailored to nursing staff have demonstrated improved protective behaviors and sustained knowledge of radiation safety over time.

This study examined how well core radiation protection principles time, distance, and shielding are applied in practice, and the findings point to important shortcomings in everyday behaviour. While most nurses reported using basic protective measures and personal dosimeters, adherence was inconsistent across experience levels, and a small minority reported using no protection at all. Such patterns suggest that the issue extends beyond simple lack of knowledge to weaknesses in habit formation, safety culture, and local enforcement of protocols. Consistent with previous work showing that healthcare staff often understand radiation safety guidelines but struggle to apply them in routine clinical conditions, the present results highlight a persistent knowledge–practice gap (Rodrigues et al., 2024; Alotaibi et al., 2015; Jeyasugiththan et al., 2023). Potential contributors include limited formal training, discomfort or inconvenience associated with protective equipment, competing clinical demands, and inconsistent reinforcement of safety rules by institutions.

The observation that a substantial proportion of nurses were unfamiliar with occupational dose limits is particularly concerning, as dose awareness is fundamental for informed decision-making and for calibrating protective behaviours (Antunes-Raposo et al., 2022). Inadequate understanding of dose limits, especially among less experienced staff, points to weaknesses in both initial education and ongoing in-service training, and may be compounded by the absence of clear workplace reminders (such as posted dose charts near workstations). To close this gap, institutions should embed mandatory teaching on dose limits and practical protection strategies into orientation programmes, reinforce this content through regular refresher training, and support it with visible prompts in clinical areas. Strengthening these structural and educational elements is essential if theoretical knowledge about radiation risks is to be reliably translated into consistent, safe practice for both staff and patients.

International bodies such as the International Atomic Energy Agency (IAEA) and International Commission on Radiological Protection (ICRP) emphasize that occupational radiation protection should be ensured through formal education and regular training, clear institutional responsibility, and monitoring of staff doses within established dose limits (International Atomic Energy Agency, 2018).

The European Union (EU) Basic Safety Standards Directive (2013/59/Euratom) and related legislation similarly require employers to implement radiation protection programmes, classify and monitor exposed workers, and keep effective doses below 20 mSv per year under normal conditions (European Union, 2013).

A key limitation of this study is the sample size, as only 233 participants were enrolled. This smaller sample may have reduced the statistical power of some chi-square tests, particularly for subgroup analyses, increasing the possibility of type II errors and meaning that some real associations may not have been detected. This shortfall may have reduced the study’s ability to detect significant effects and limit the generalizability of the results. The primary reason for the reduced sample was the limited data collection period, which restricted recruitment and prevented reaching the intended target. However, the study’s findings highlight the level of awareness of radiation safety procedures among nurses in the NM department. Future studies should incorporate objective measures of radiation safety practices, such as observational checklists or dosimetric monitoring, to corroborate self-reported data and strengthen the evidence base with extended collection timelines to increase the sample size and strengthen the statistical validity of the findings.

## Conclusions

Radiation safety is a critical aspect of nursing practice in NM departments, where healthcare professionals are routinely exposed to ionizing radiation. This study highlights that while nurses generally possess foundational awareness of radiation protection, there are evident shortcomings in advanced knowledge, training, and consistent application of safety measures. The results indicate that nurses’ awareness of radiation safety procedures in the NM department varies significantly. A large proportion of participants demonstrated moderate to good theoretical knowledge of basic radiation protection principles; however, gaps were observed in their practical application. The lack of formal education among many nurses and inconsistent institutional support further underscores the need for systemic improvements.

To ensure a safe working environment and uphold patient safety, healthcare institutions must prioritize ongoing, practical radiation safety education. A strong safety culture, reinforced by accessible training and strict protocol enforcement, will empower nurses to confidently and effectively mitigate radiation risks in their daily practice.

### Recommendation

Hospitals and healthcare institutions should establish regular, mandatory radiation safety training tailored specifically for nurses in nuclear medicine. It is essential to implement structured, continuous education programs to improve nurses’ radiation safety awareness. Additionally, integrating online modules and mandatory workshops allows flexible access to updated guidelines and protocols, while simulations provide hands-on experience in applying safety principles in realistic clinical scenarios. Moreover, continuing professional development (CPD) opportunities, including workshops and accredited training sessions focused on specialized radiation safety programs, can enhance nurses’ awareness of radiation risks and safety practices. Furthermore, hospital administrations must consistently enforce radiation safety protocols and conduct regular audits to ensure compliance across all staff levels. Finally, academic institutions should incorporate comprehensive radiation protection modules into nursing education, ensuring that graduates enter the workforce with essential safety knowledge.

## Supplemental Information

10.7717/peerj.21109/supp-1 Supplemental Information 1 Raw data

10.7717/peerj.21109/supp-2 Supplemental Information 2 STROBE checklist

10.7717/peerj.21109/supp-3 Supplemental Information 3 Supplemental Materials

## List of abbreviations

NM: Nuclear Medicine
PET: Positron Emission Tomogrhapy
SPECT: Single Photon Emission Computed Tomography
SFDA: Saudi Food and Drug Authority
NRRC: Nuclear and Radiological Regulatory Commission
ALARA: As Low As Reasonably Achievable
mSv: millisievert
SPSS: Statistical Package for Social Sciences
PPE: Personal Protective Equipment
IAEA: International Atomic Energy Agency
ICRP: International Commission on Radiological Protection
EU: European Union
CPD: continuing professional development

## Additional Information and Declarations

### Data Availability

The following information was supplied regarding data availability: The raw data are available in the Supplemental File.
