# Supplementary figures and images for: Assessment of radiation safety awareness among nurses in nuclear medicine departments
Source: PeerJ. 2026 Apr 17;14:e21109. doi: 10.7717/peerj.21109 (PMC13094554; doi:10.7717/peerj.21109)

Supplementary materials


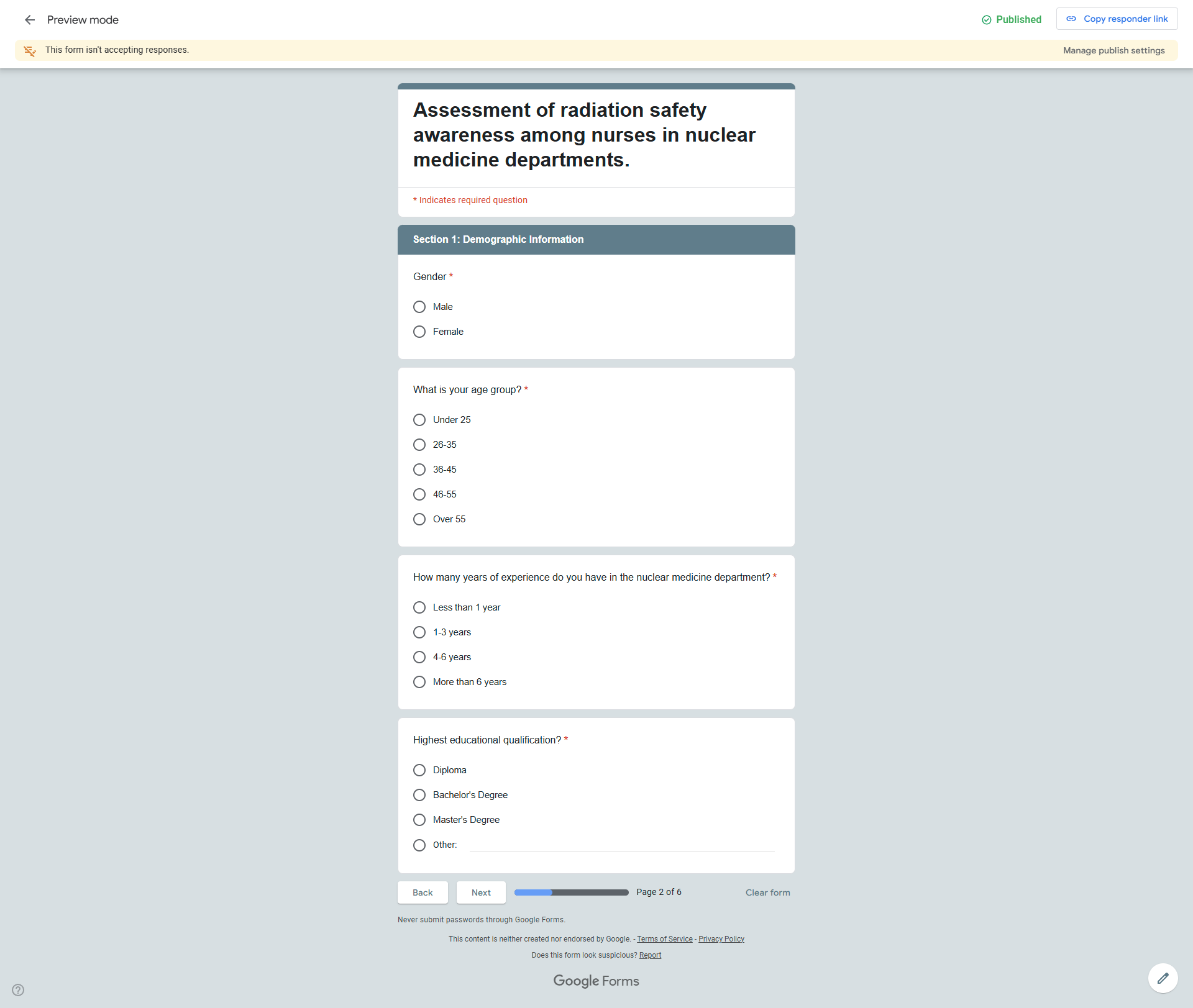

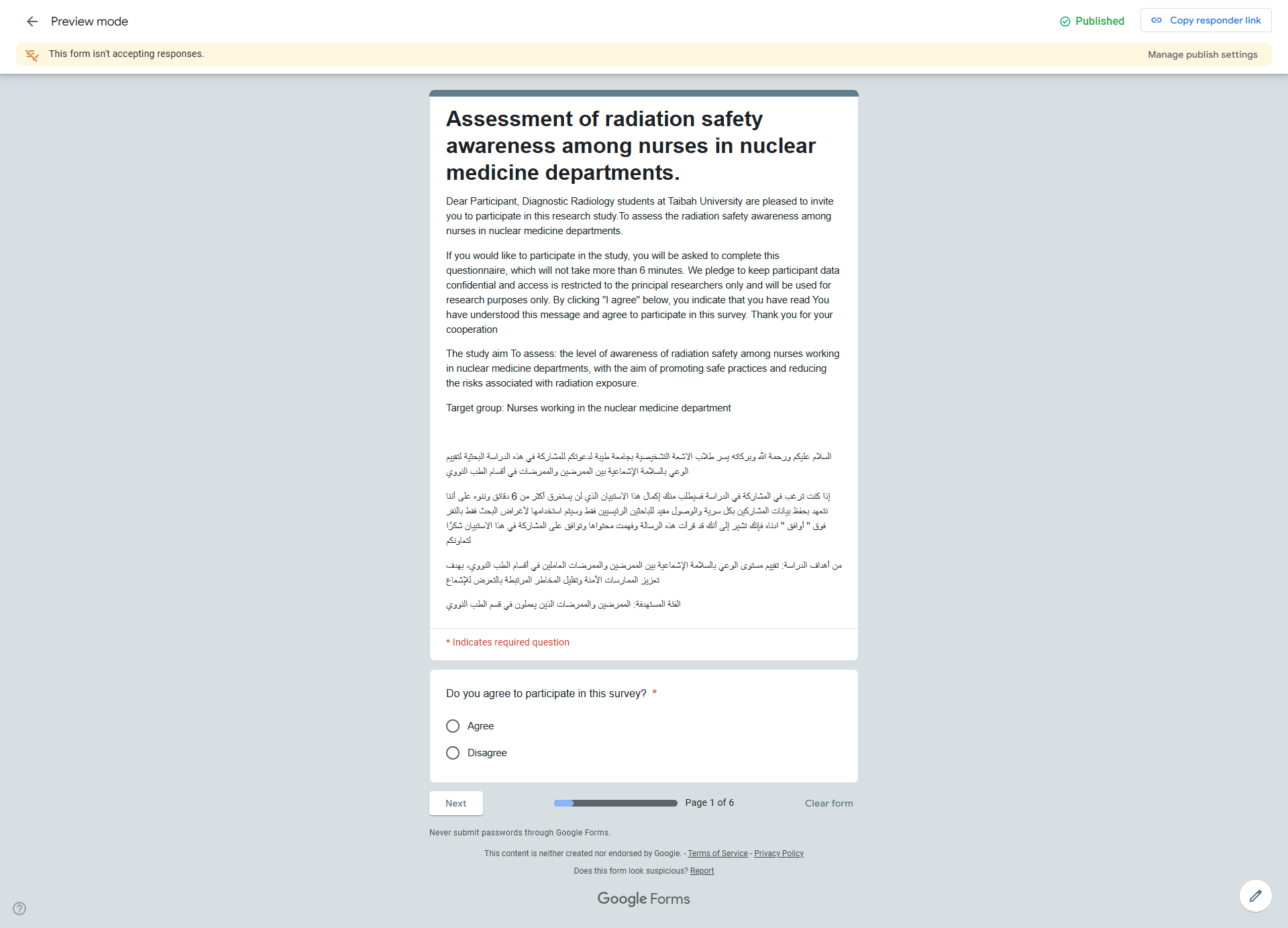


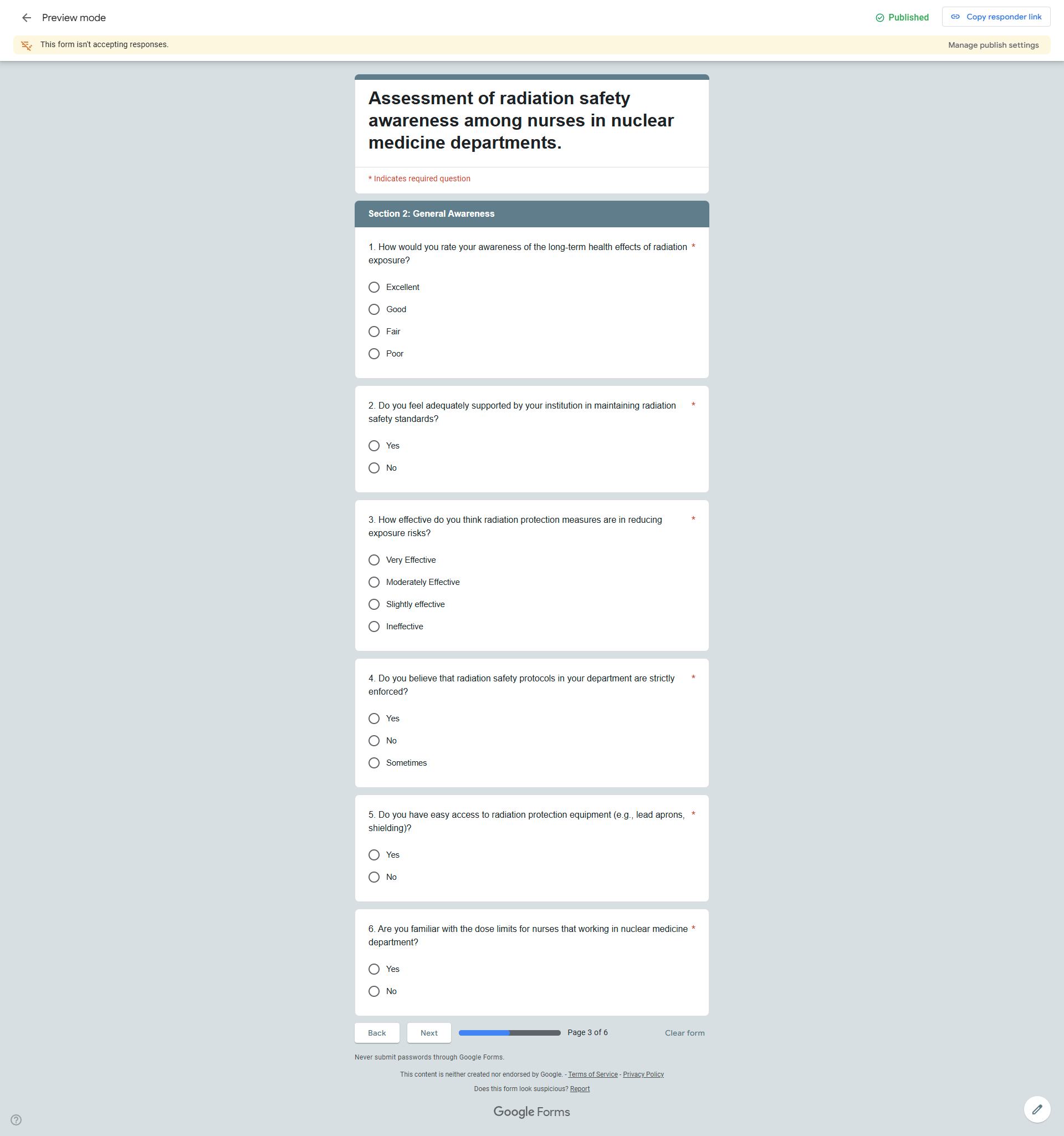

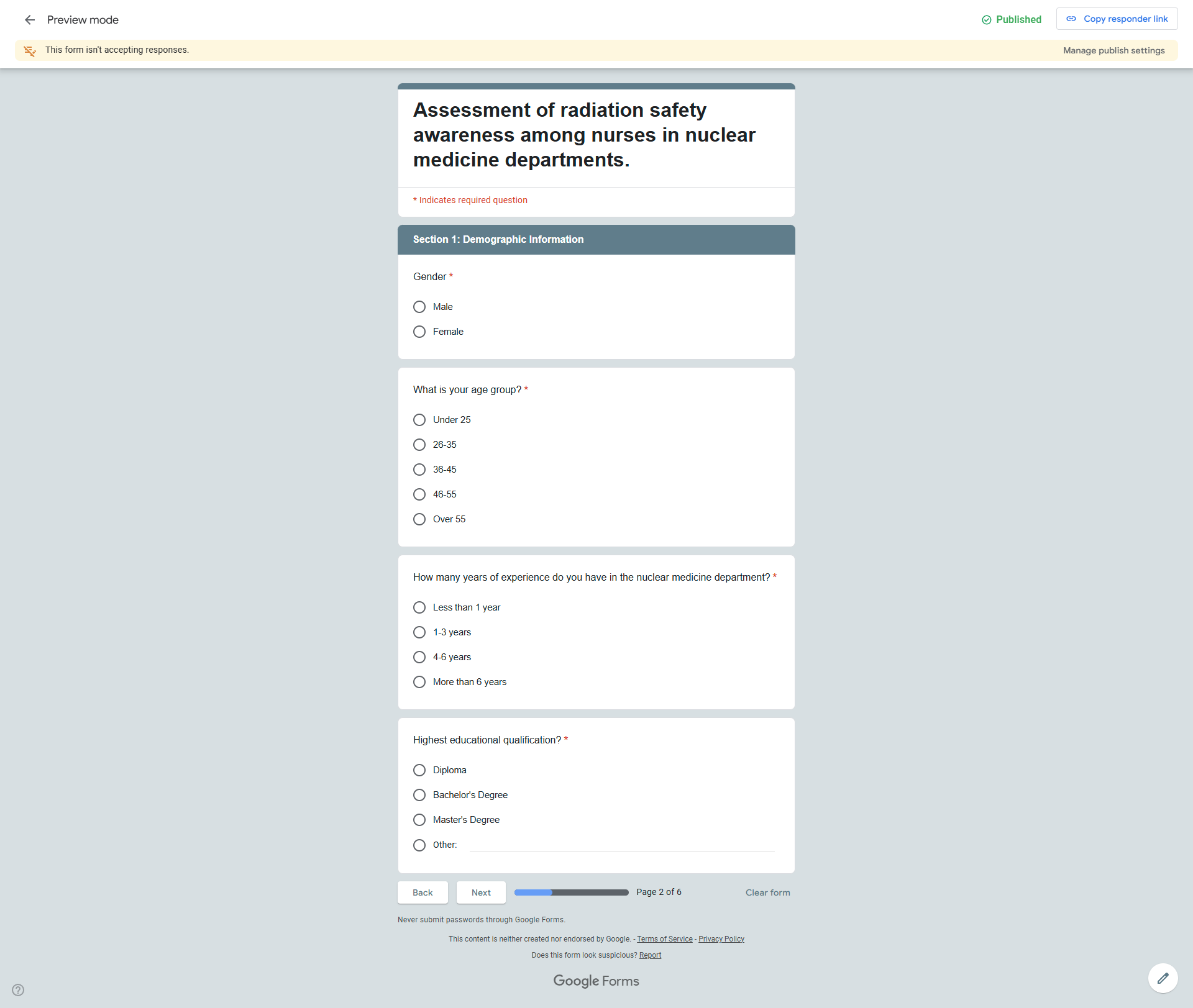


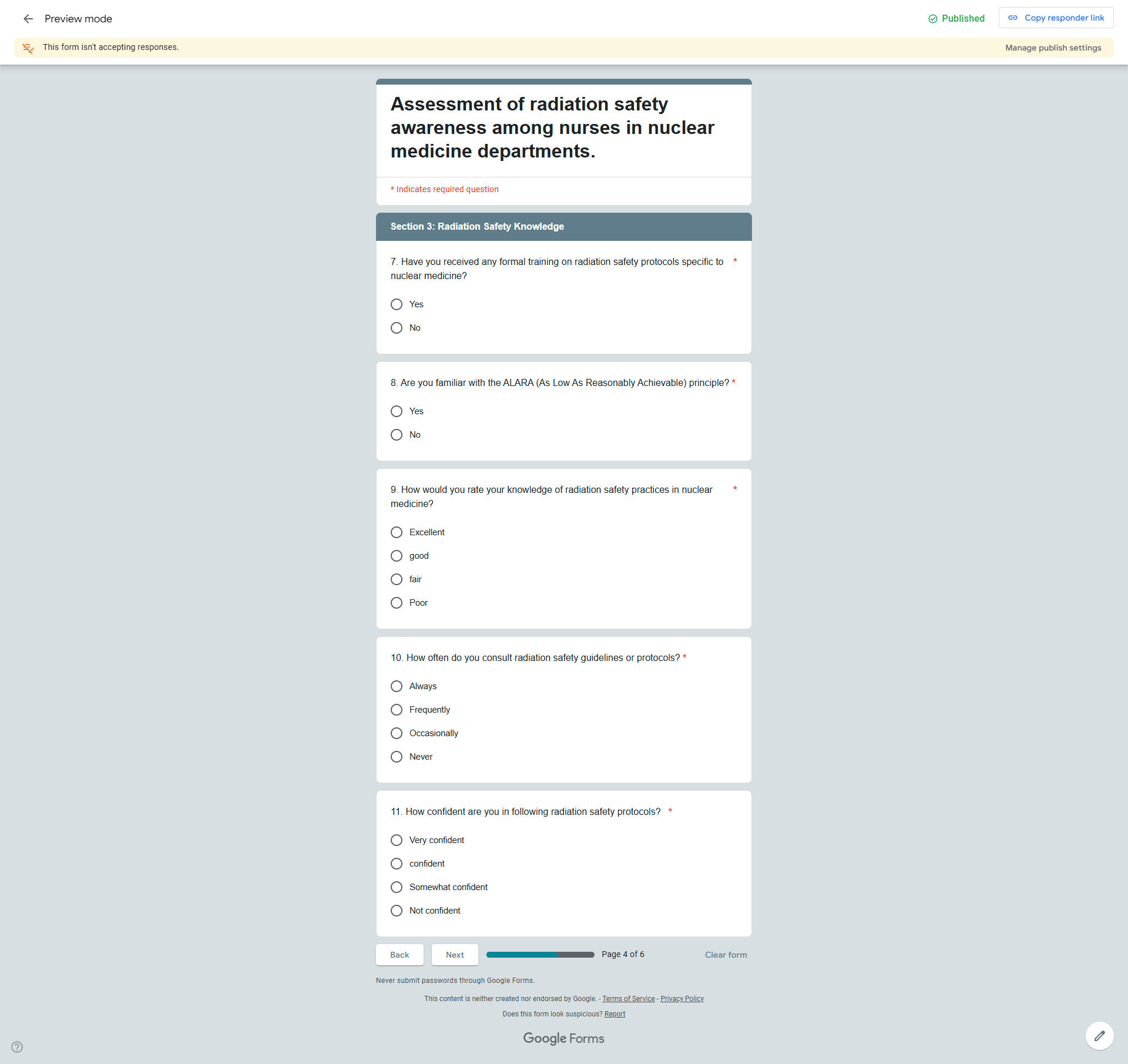

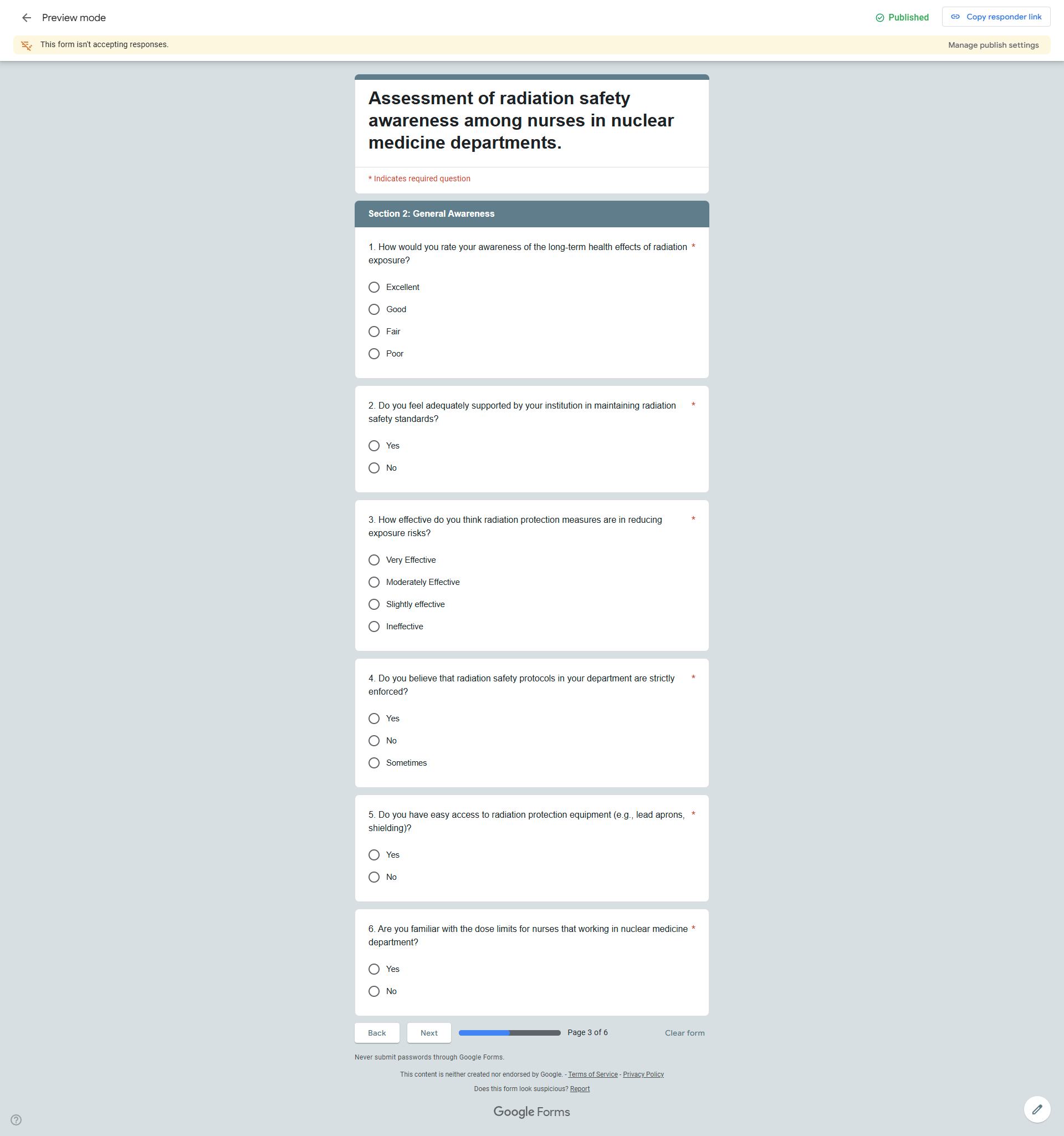


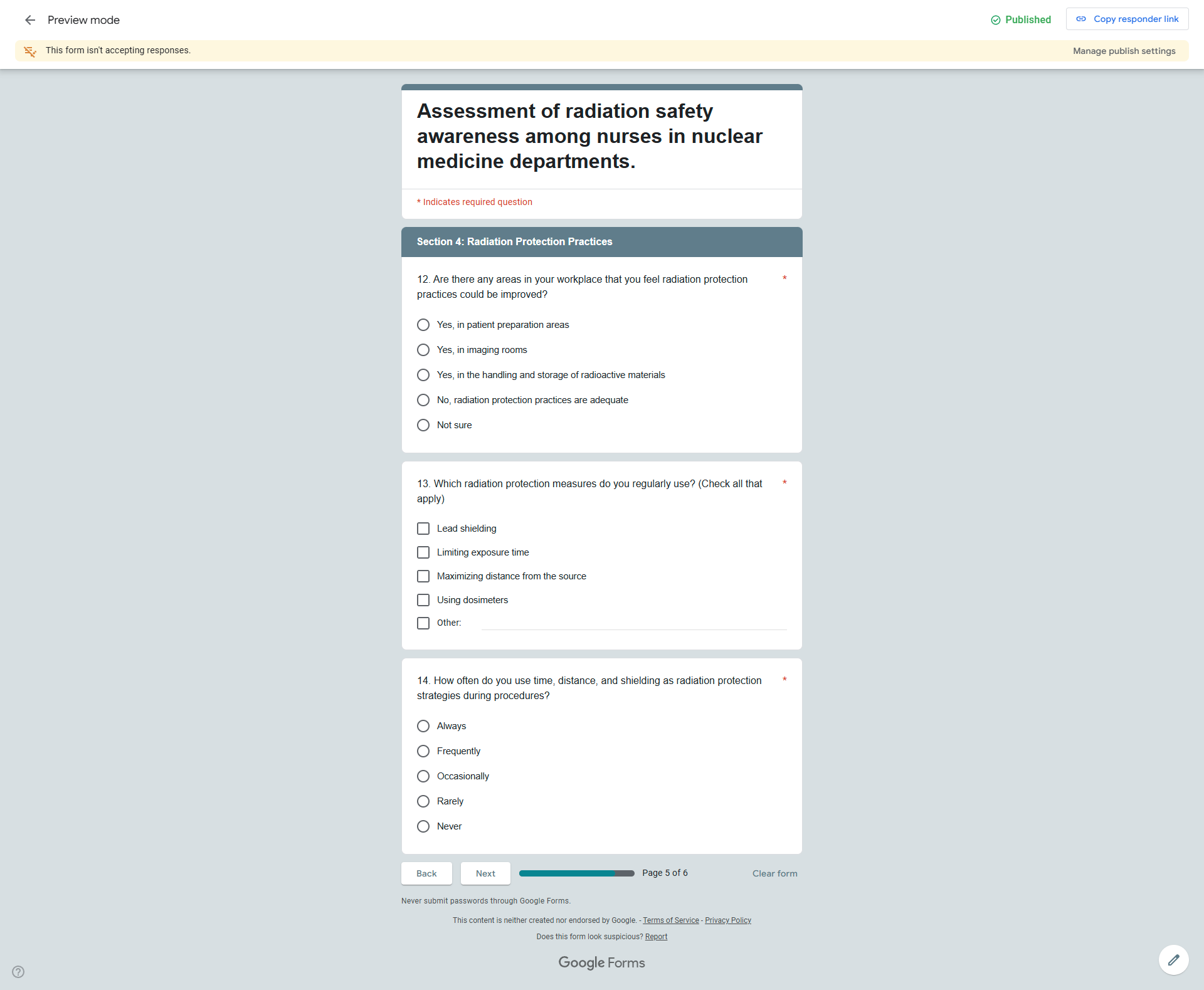

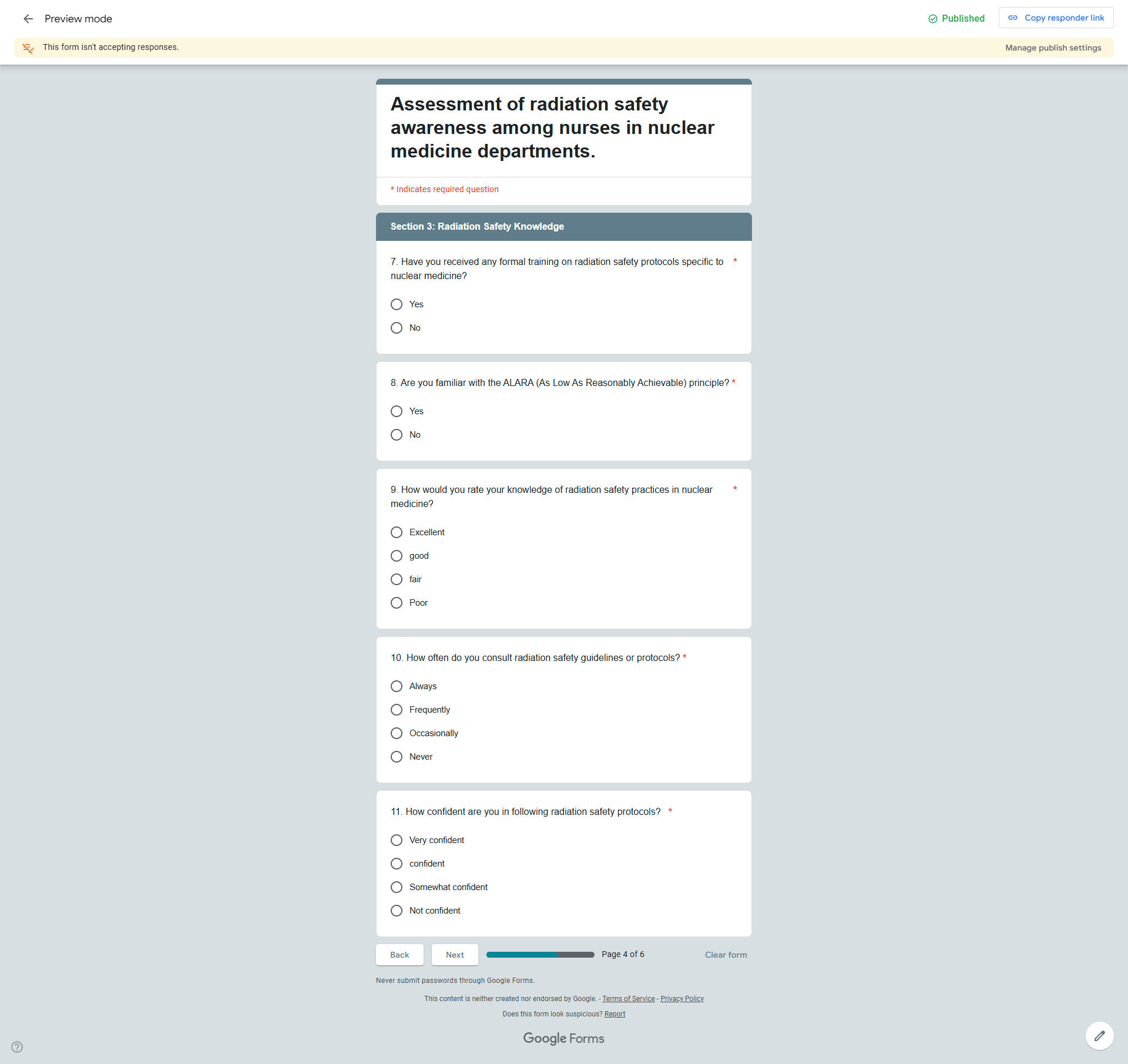


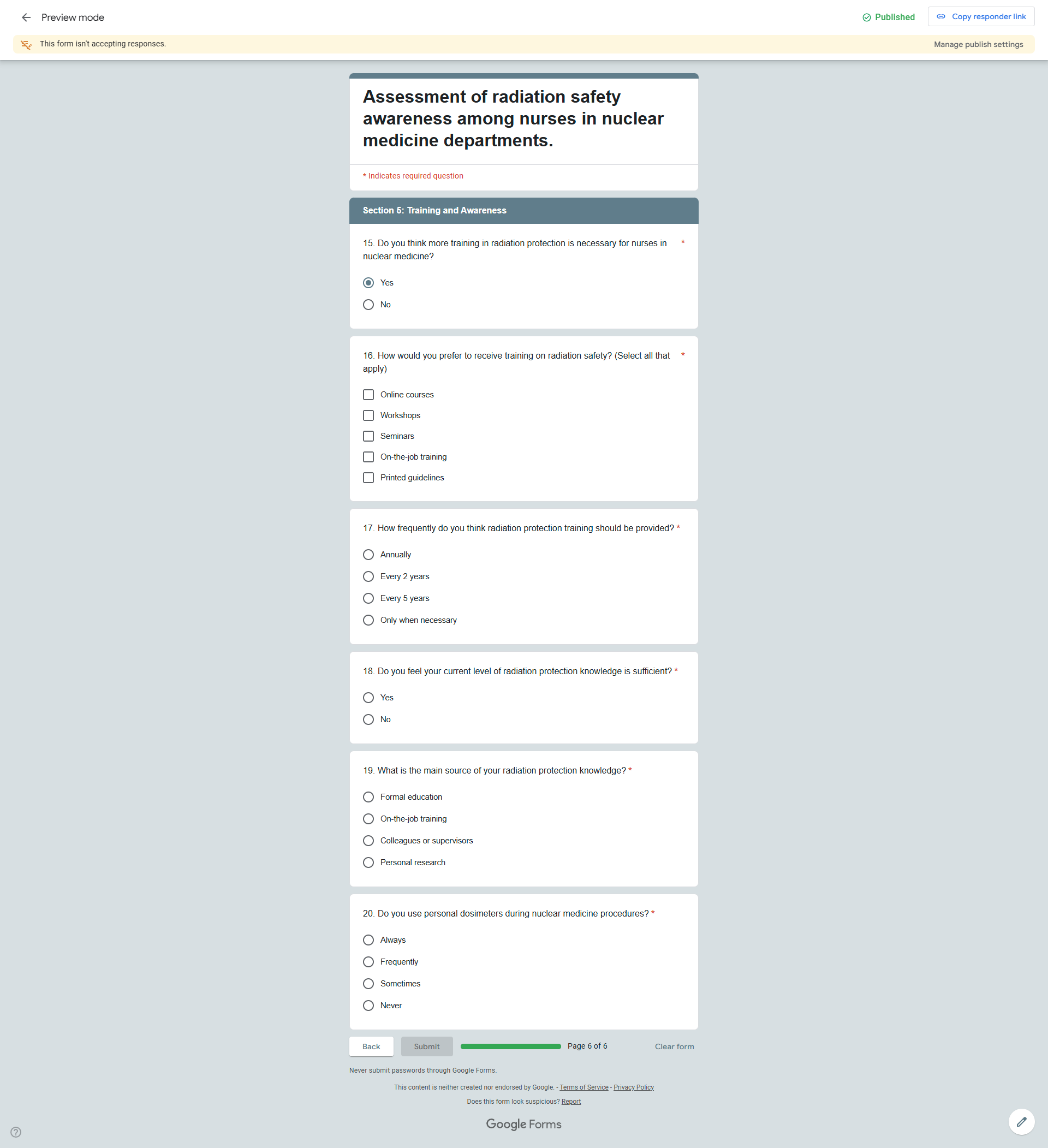

Supplement: Supplemental Information 3 [file peerj-14-21109-s003.docx]
